# Association of Dietary Calcium Intake With Bone Health and Chronic Diseases: Two Prospective Cohort Studies in China

**DOI:** 10.3389/fnut.2021.683918

**Published:** 2021-12-24

**Authors:** Xiaoyu Guo, Jian Gao, Xing Meng, Jiemei Wang, Ziwei Zhang, Qingrao Song, Ke Hu, Changhao Sun, Ying Li

**Affiliations:** National Key Discipline, Department of Nutrition and Food Hygiene, School of Public Health, Harbin Medical University, Harbin, China

**Keywords:** dietary calcium intake, chronic disease, cohort study, dietary reference intakes (DRI), bone mineral density (BMD)

## Abstract

**Background:** Calcium is an essential element in our diet and the most abundant mineral in the body. A high proportion of Chinese residents are not meeting dietary calcium recommendations. The purpose of this study was to investigate the relationship between calcium intake and the health of residents in two longitudinal studies of Chinese residents.

**Methods:** This study used nationally representative data from the Harbin Cohort Study on Diet, Nutrition, and Chronic Non-communicable Disease Study (HDNNCDS) and China Health Nutrition Survey (CHNS), including 6,499 and 8,140 Chinese adults, respectively, who were free of chronic diseases at recruitment, with mean values of 4.2- and 5.3-year follow-up. Cox's proportional-hazards regression was conducted to explore the relationship between dietary calcium intake and the incidence of obesity, type 2 diabetes, hypertension, and cardiovascular disease (CVD) with adjustment for covariates.

**Results:** Calcium intakes were 451.35 ± 203.56 and 484.32 ± 198.61 (mean ± SD) mg/day in HDNNCDS and CHNS. After adjusting the covariates, the relationship between dietary calcium intake and bone mineral density (BMD) was not statistically significant (*p* = 0.110). In the multivariate-adjusted Cox's proportional-hazards regression model, dietary calcium intakes were inversely associated with obesity incidence in both cohorts (HR [95% CI]: 0.61 [0.48–0.77] and *p* trend < 0.001 in fixed-effects model); nevertheless, there was no correlation between dietary calcium intake and the risk of type 2 diabetes (*p* trend = 0.442 and 0.759) and CVD (*p* trend = 0.826 and 0.072). The relationship between dietary calcium intake and the risk of hypertension in the two cohorts was inconsistent (*p* trend = 0.012 and 0.559). Additionally, after further adjusting the vegetable intake in the original multivariate model, both cohorts found no association between dietary calcium intake and the risk of developing obesity (*p* trend = 0.084 and 0.444).

**Conclusions:** Our data suggest that the current calcium intake of Chinese residents was inversely associated with obesity, which may be related to consumption of vegetables. Meanwhile, the current calcium intake does not increase the risk of type 2 diabetes, CVD, and bone health burden. This research suggested that the Chinese current calcium intake level may have met the needs of the body.

## Introduction

Calcium is an essential nutrient in the human body. Adequate calcium intake is essential for the normal growth and development of bones and the necessary physiological functions (1). Although short-term dietary calcium deficiency will not lead to a significant decrease in blood calcium levels, long-term dietary calcium deficiency will eventually lead to bone weakness and easy fracture (2, 3). Recent evidence suggests that calcium may also have non-skeletal effects. To date, numerous epidemiological studies and meta-analyses have found that there seems to be a close link between dietary calcium deficiency and the prevalence of chronic disease (4, 5).

Despite this, according to the China Nutrition and Health Status Survey, the average dietary calcium intake of Chinese residents in 2002 and 2012 was 389 and 364 mg/d, respectively, which is only about 50% of the recommended intake (6, 7). The current reference recommended intake of dietary calcium for Chinese residents is 800 mg/day (8). Notably, the Chinese recommended intake of calcium has been formulated based primarily on reference to the US standard, which is only slightly lower than the US at the time of formulation given the differences in bone mass between the Chinese and US populations (9). Due to ethnic differences in calcium absorption, metabolism, and dietary habits, the reference intake based on the western population may not be fully applicable to the Chinese population (10, 11). Moreover, a meta-analysis including 11 calcium balance studies showed that the calcium requirement for Chinese adults approximately ranged between 400 and 500 mg/d (9), which was close to the average dietary calcium intake of Chinese residents. Given the limitation and inconsistency of evidence, the relationship between dietary calcium and health in the normal population remains unclear. Meanwhile, most previous studies on the association between calcium intake and chronic disease have been conducted in Western populations (12), with only limited evidence in Chinese, despite a steadily increasing prevalence of chronic disease over the past decade.

Therefore, it is necessary to study the relationship between dietary calcium intake and bone health and non-bone health on the premise that the calcium intake of Chinese residents is generally lower than the recommended intake. In this research, two prospective cohorts from the Harbin Cohort Study on Diet, Nutrition, and Chronic Non-communicable Disease Study (HDNNCDS) and the China Health and Nutrition Survey (CHNS) were used to investigate the association of calcium intake with bone health and the incidence of chronic diseases including obesity, type 2 diabetes, hypertension, and cardiovascular disease (CVD).

## Methods

### Study Populations

Harbin Cohort Study on Diet, Nutrition, and Chronic Non-communicable Disease Study is an ongoing prospective cohort study launched in 2010. The study's design and implementation have been described (13). In brief, at baseline, 9,734 participants aged 20–74 years old had lived in Harbin for more than 2 years, without type 1 diabetes and malignancies were recruited. From 2015 to 2016, a total of 8,913 participants finished the first follow-up survey with a response rate of 91.6%. In this study, first, we excluded the following individuals: those who were taking vitamin D supplementation or calcium gluconate injection at either the baseline or the follow-up; those with dietary restriction for diseases or weight loss; those with extremely high or low total energy intake (>4,000 or <800 kcal); and also those with more than 10 blank items in the dietary questionnaire. Furthermore, when analyzing the relationship between calcium and obesity, we further exclude those with obesity (self-reported or diagnosed based on BMI) at baseline; Similarly, when analyzing the relationship between calcium and hypertension, CVD, and type 2 diabetes, we also excluded participants with these diseases at baseline, respectively (Figure S1). The study was carried out at Harbin Medical University in Harbin and approved by the ethics committee of Harbin Medical University and conducted in accordance with the Declaration of Helsinki (ChiCTRECH-12002721).

China Health and Nutrition Survey is a nationwide ongoing longitudinal prospective cohort study of nine waves (1989, 1991, 1993, 1997, 2000, 2004, 2006, 2009, and 2011). A multistage, random cluster process was used to obtain the representative samples of 228 communities from nine diverse provinces in China, and new participants have been recruited as replenishment samples since 1997 (14). The overall response rate was 93%, based on those who were followed up at least once.

As hematological specimens were only collected and detected in the 2009 survey, to accurately measure the occurrence of chronic diseases, only participants who provided hematological specimens during the follow-up period in 2009 and complete information at any of the four baseline surveys in 1997, 2000, 2004, and 2006 were included in this study. After excluding those with implausible or missing values for the intake of dietary, or total energy intake and dietary calcium (the upper and lower 0.5% of the intakes), and also those with type 2 diabetes, hypertension, obesity, and CVD at baseline, 6,499 participants were included in the final analyses. The survey protocols, instruments, and the process for obtaining informed consent were approved by the Institutional Review Committees of the University of North Carolina at Chapel Hill, NC, USA, and the China National Institute of Nutrition and Food Safety at the Chinese Center for Disease Control and Prevention, Beijing, China. All participants provided written informed consent prior to the surveys.

### Assessment of Diet

In the HDNNCDS, baseline dietary intake was evaluated using a validated food frequency questionnaire (FFQ) consisting of 103 food items divided into 14 food groups to assess habitual dietary intakes over the past year. To evaluate the validity and repeatability of the FFQ, we have conducted a small-scale study in 2009 and found that FFQ used in HDNNCDS was a reliable method for assessing dietary intake (13). In this study, each participant was asked about the frequency and quantity of each food item that they have consumed by trained nutrition professionals. Daily nutrient intake from the diet was calculated in accordance with the Chinese Food Composition Table (FCT). For the CHNS, to assess individual diet in each survey, the researchers repeatedly performed a 24-h review of individual diet and measured household oil and condiment consumption for three consecutive days. We calculated nutrient intakes as the nutrient content of standard portion size of 100 g multiplied by the consumption of each food item and used China FCTs were utilized to obtain the nutrient intake data of each participant.

### Basic Information Collection and Biochemical Assessments

In both cohorts, all the participants were interviewed face-to-face by trained personnel using a structured questionnaire to collect demographic and lifestyle information, mainly including physical activity level, educational status, smoking, drinking status, family history of diseases, and medical history. At the same time, anthropometric indices, body weight, and height were taken in agreement with standardized procedures. Blood pressure was measured 3 times on the right arm, with a standard mercury sphygmomanometer after a 10-min rest before measurement, and the mean values were used for statistical analysis.

Blood samples, including fasting and postprandial (2 h after drinking 75 g of water containing glucose), were collected by professional nurses at the follow-up survey in both HDNNCDS and CHNS. Additionally, in HDNNCDS, blood samples were also collected at the baseline survey. Serum fasting blood glucose and 2-h glucose were measured using an automatic analyzer (Hitachi 7100, and Hitachi 7100, Tokyo, Japan, respectively). In addition, in HDNNCDS, the serum level of 25OHD_3_ was measured based on UPLC (Waters Corporation, Milford, MA, USA) (15).

During the follow-up period of HDNNCDS, the bone mineral density (BMD) of 1,640 subjects without fractures and bone metabolic diseases was measured by dual-energy X-ray absorptiometry Hologic QDR 4500 (DEXA). Measurements were taken at the following sites: femoral neck, intertrochanter, ward triangle, greater trochanter, and total hip. All measurements were collected by trained staff using standard methods.

### Outcome Identification

Obesity was defined as BMI ≥ 28 kg/cm^2^, according to the guidelines for prevention and control of overweight and obesity in Chinese adults (16). Type 2 diabetes at follow-up was defined by ADA criteria as FBG ≥ 7.0 mmol/L, and/or 2-h PG ≥ 11.1 mmol/L, and/or receiving treatment for type 2 diabetes (17). Diagnostic criteria for hypertension defined by evidence-based guidelines for the management of high blood pressure in adults were as follows: SBP ≥ 140 mmHg, and/or DBP ≥ 90 mmHg, and/or receiving treatment for hypertension (18). CVD was diagnosed by self-reported information of the history of stroke and/or myocardial infarction (19).

### Statistical Analysis

Dietary calcium intakes were energy-adjusted using the residuals calculated from the linear regression for data analysis. Participants were categorized into quartile groups according to their calcium intake levels at the baseline from the lowest intake (quartile 1) to the highest intake (quartile 4). The mean ± SD was expressed for each continuous data. Category value was expressed as a proportion. Using a general linear model to compare differences in BMD between different dietary calcium intake levels with adjustment for age, sex, BMI, alcohol consumption, smoking, education level, physical activities, dietary total energy, and menopause in the case of women, Cox's proportional-hazards regression was performed to analyze the association of calcium intake and the incidence of obesity, type 2 diabetes, hypertension, and CVD with adjustment for covariates. We used restricted cubic spline (RCS) as a tool available for characterizing dose–response associations between dietary calcium intake with incidence of obesity and hypertension, using 3 knots at pre-specified locations according to the percentiles of the distribution of dietary calcium, the 5, 50, and 95th percentiles. The reference value for calcium intake was chosen to be the median value (20). Stratified analysis was conducted among adults of different genders and ages. Meta-analysis was performed using comprehensive meta-analysis (CMA) V3.0 software (https://www.meta-analysis.com/) with fixed-effects model, and other statistical analyses were performed using R 3.2.5 (http://www.r-project.org/) and SPSS software version 22.0 (SPSS; Beijing Stats Data Mining Co. Ltd). A two-tailed value of *p* < 0.05 was considered significant.

## Results

### Baseline Characteristics of Subjects Based on Calcium Intake

Table 1 showed the baseline characteristics of the population of these two studies, which were described by quartiles of dietary calcium intake. With the increase in dietary calcium intake, in both studies, the age and proportion of women gradually increased. The individuals in the highest quartile of dietary calcium intake were less likely to smoke and drink alcohol than those in the lowest quartile. High calcium intake was associated with high education level. In addition, there were significant differences in physical activity levels among the four groups in two cohort studies.

**Table 1:** Baseline characteristics according to quartiles of dietary calcium intake at baseline of the population in two cohort studies.

**Characteristics**	**Dietary calcium intake**				***P*** **^b^**
	**Q1^a^**	**Q2**	**Q3**	**Q4**	
**HDNNCDS**					
Participants (*n*)	2,036	2,034	2,036	2,034	
Dietary Ca intake^c^ (mg/d)	256.62 ± 60.93	377.51 ± 26.89	484.79 ± 36.26	704.77 ± 149.87	
Age (years)	51.13 ± 9.23	51.45 ± 9.46	52.36 ± 9.55	52.65 ± 9.34	<0.01
Female (%)	56.2	66.9	67.8	71.5	<0.01
Smoking (%)					<0.01
Current	22.8	15.0	14.0	12.6	
Ever	4.9	2.8	3.9	2.5	
Never	72.4	82.2	82.1	84.9	
Alcohol consumption (%)	39.5	30.9	32.4	28.5	<0.01
Education (%)					0.01
<9 years	36.1	33.8	30.4	29.2	
10–12 years	30.6	32.1	34.7	35.4	
>12 years	27.5	27.4	28.7	28.9	
Physical activity (%)					<0.01
Light	77.7	82.0	85.0	84.8	
Middle	19.2	17.1	13.5	13.9	
Heavy	3.1	0.9	1.5	1.3	
**CHNS**					
Participants (n)	1,624	1,626	1,624	1,625	
Dietary Ca intake^c^ (mg/d)	283.08 ± 125.12	395.30 ± 129.36	528.21 ± 134.56	795.50 ± 184.20	
Age (years)	42.00 ± 12.90	43.20 ± 13.03	43.92 ± 13.10	44.07 ± 12.92	<0.01
Female (%)	49.7	55.5	56.2	54.6	0.01
Smoking (%)					<0.01
Current	34.6	29.6	30.2	27.3	
Ever	0.4	0.6	0.6	0.9	
Never	65.0	69.8	69.2	71.8	
Alcohol consumption (%)	34.1	29.6	30.7	27.0	<0.01
Education (%)					<0.01
<9 years	27.8	27.6	27.6	26.3	
10–12 years	55.8	54.0	53.3	52.1	
>12 years	14.7	15.6	16.3	18.6	
Physical activity (%)					<0.01
Light	19.3	21.6	22.7	25.8	
Middle	10.3	12.0	13.7	14.0	
Heavy	42.1	31.1	26.4	25.2	

a *All values represent means ± SD for continuous variables and proportions for categorical variables*.

b *Differences between four groups were tested by using ANOVA and chi-squared test for continuous and categorical variables, respectively*.

c *Energy-adjusted by using the residual method*.

### Associations Between Calcium Intake and BMD

Due to the lack of bone health data in CHNS, research on the relationship between dietary calcium and bone health was only conducted in HDNNCDS. After adjusting for covariates, there was no significant difference in BMD of the femoral neck, femoral intertrochanteric, ward triangle, greater trochanter, and total hip among different dietary calcium intake groups (Table 2).

**Table 2:** Comparison of BMD in different dietary calcium intake groups.

**Variables**	**Dietary calcium intake (mg/d)**				** $p_{\text{trend}}^{\text{b}}$ **
	**Q1^a^**	**Q2**	**Q3**	**Q4**	
Participants (*n*)	409	410	411	410	
Bone mineral density (g/cm^2^)					
Femoral neck	0.75 ± 0.01	0.75 ± 0.01	0.76 ± 0.01	0.75 ± 0.01	0.523
Intertrochanter	0.93 ± 0.01	0.95 ± 0.01	0.97 ± 0.01	0.95 ± 0.01	0.168
Ward triangle	0.54 ± 0.01	0.54 ± 0.02	0.57 ± 0.02	0.56 ± 0.01	0.292
Greater trochanter	0.59 ± 0.01	0.60 ± 0.01	0.62 ± 0.01	0.60 ± 0.01	0.158
Total hip	0.80 ± 0.01	0.82 ± 0.01	0.83 ± 0.01	0.81 ± 0.01	0.110

a *Continuous variables were shown as means ± standard error*.

b *Model were adjusted by age, sex, BMI, alcohol consumption, smoking, education, physical activities, dietary total energy, and menopause in case of women. BMI, body mass index*.

### Association Between Calcium Intake and Chronic Disease

**Figure 1:**
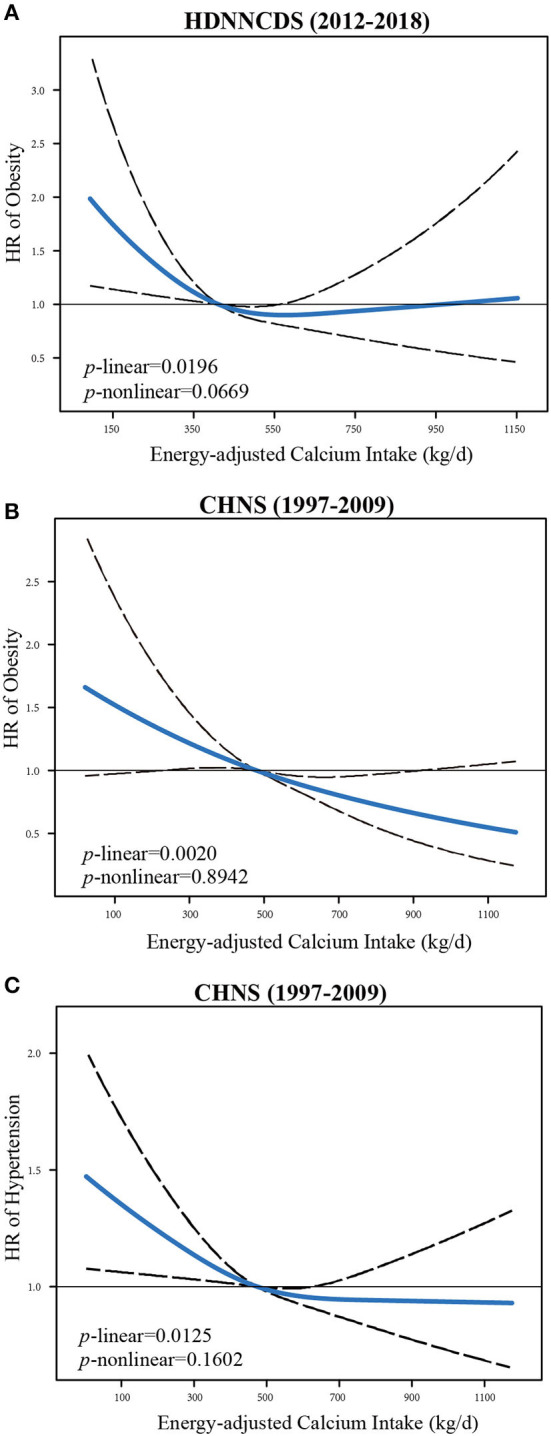
Association between dietary calcium intake and the risk of obesity in HDNNCDS **(A)** and CHNS study **(B)**, and the risks of obesity and hypertension in CHNS study **(C)**, allowing for linear effects, with 95% CIs. The models with 3 knots RCS for calcium intake adjusting for age, sex, BMI, alcohol consumption, smoking, education, physical activities, dietary total energy, and menopause in case of women. Curves showed HRs of obesity or hypertension compared with the chosen reference medians of calcium intakes. BMI, body mass index; HRs, hazard risk.

Table 3 showed the association between calcium intake and four common chronic diseases in HDNNCDS study. During 5.3-year of follow-up, we identified 506 incident cases of obesity, 1,378 incident cases of hypertension, 614 incident cases of type 2 diabetes, and 898 incident cases of CVD. By comparing quartile 4 with quartile 1, we observed that obesity risk decreased with dietary calcium intake (HR: 0.58, 95% CI: 0.40–0.83, *p* for trend = 0.015). In addition, we found no appreciable association between dietary calcium intake and the risk of hypertension, type 2 diabetes, and CVD. We modeled the relationship between dietary calcium intake and obesity risk using the RCS model with 3 knots (Figure 1A). There was a significant linear dose–response association visually, but not non-linear relationship (*p* for linear = 0.020; *p* for non-linear = 0.067).

**Table 3:** Hazard ratios (HRs) and 95% CIs of chronic disease according to energy-adjusted dietary calcium intake in HDNNCDS (2010–2016).

**Variables**	**Energy-adjusted dietary calcium intake**				***p*** **_trend_**
	**Q1**	**Q2**	**Q3**	**Q4**	
**Obesity**					
Intake (mg/d)	294.81 ± 124.12	342.72 ± 125.76	464.67 ± 128.95	720.74 ± 210.09	
Case/N	176/1,650	112/1,654	126/1,652	92/1,650	
Model 1^a^	Reference	0.68 (0.49–0.95)	0.77 (0.56–1.07)	0.58 (0.41–0.83)	0.006
Model 2^b^	Reference	0.67 (0.47–0.95)	0.78 (0.56–1.10)	0.60 (0.42–0.87)	0.016
Model 3^c^	Reference	0.65 (0.46–0.93)	0.76 (0.54–1.07)	0.58 (0.40–0.83)	0.015
**Hypertension**					
Intake (mg/d)	255.78 ± 59.74	379.47 ± 27.40	486.59 ± 36.75	704.54 ± 144.90	
Case/N	356/1,332	360/1,336	342/1,334	320/1,332	
Model 1	Reference	1.05 (0.85–1.29)	0.97 (0.78–1.20)	0.90 (0.72–1.11)	0.224
Model 2	Reference	1.06 (0.85-1.33)	1.01 (0.81-1.27)	0.95 (0.76-1.19)	0.539
Model 3	Reference	1.04 (0.83–1.30)	0.99 (0.79–1.24)	0.95 (0.76–1.19)	0.559
**Type 2 diabetes**					
Intake (mg/d)	256.41 ± 60.75	376.10 ± 26.50	483.60 ± 36.92	704.12 ± 149.67	
Case/*N*	158/1,874	154/1,876	62/1,876	140/1,874	
Model 1	Reference	0.99 (0.73–1.36)	1.02 (0.75–1.40)	0.88 (0.64–1.22)	0.428
Model 2	Reference	1.00 (0.73–1.38)	1.04 (0.76–1.43)	0.88 (0.63–1.23)	0.444
Model 3	Reference	1.02 (0.73–1.41)	1.05 (0.76–1.44)	0.88 (0.63–1.23)	0.442
**Cardiovascular disease**					
Intake (mg/d)	266.22 ± 76.05	399.74 ± 27.54	503.26 ± 37.15	731.80 ± 164.39	
Case/*N*	208/1,316	208/1,314	246/1,312	236/1,316	
Model 1	Reference	0.98 (0.75–1.29)	1.08 (0.83–1.40)	1.00 (0.77–1.31)	0.887
Model 2	Reference	0.98 (0.74–1.29)	1.09 (0.83–1.42)	1.02 (0.78–1.34)	0.776
Model 3	Reference	1.03 (0.77–1.36)	1.13 (0.86–1.49)	1.02 (0.78–1.34)	0.826

a *Model 1 adjusted with age and gender*.

b *Model 2 adjusted with age, gender, BMI, alcohol consumption rate, smoking rate, physical activity, and education*.

c *Model 3 adjusted with age, gender, BMI, alcohol consumption rate, smoking rate, physical activity, education, dietary energy intake, and AHEI. BMI, body mass index; AHEI, alternative healthy eating index*.

In CHNS study, during 4.2-year of follow-up, we identified 337 incident cases of obesity, 1,228 incident cases of hypertension, 501 incident cases of type 2 diabetes, and 150 incident cases of CVD (Table 4). For the risk of obesity, compared with the lowest quartile, no statistically significant association was observed between calcium intake and obesity with adjustment for age and sex (*p* for trend = 0.050). However, after adjustment for potential confounders and related dietary factors, dietary calcium intake was associated with a depressed risk of obesity. In Model 3, the hazard ratio of the highest quartile of calcium intake was 0.63 (95% CI: 0.46–0.85, *p* for trend = 0.001). Similarly, we found the same negative correlation in the HDNNCDS study, so we used a fixed-effects model to comprehensively analyze the results of the two cohort studies. We further determined the negative correlation between dietary calcium and obesity risk. Likewise, there was no significant association between calcium and the risk of hypertension after adjusted age and sex. But in multivariate analysis, the HR (95% CI) of the highest quartile of calcium intake was 0.82 (95% CI: 0.69–0.97), compared with the lowest quartile. No statistically significant association was observed between calcium intake and type 2 diabetes, or CVD in any model. We also used RCS to model the linear or non-linear relationships between calcium intake and the risks of obesity and hypertension (Figures 1B,C). There were both significant linear dose–response associations between calcium and obesity (*p* for linear = 0.0020; *p* for non-linear = 0.8942) and hypertension (*p* for linear = 0.0125; *p* for non-linear = 0.1602).

**Table 4:** Hazard ratios (HRs) and 95% CIs of chronic disease according to energy-adjusted dietary calcium intake in CHNS (1997–2009).

**Variables**	**Energy-adjusted dietary calcium intake**				***p_trend_***
	**Q1**	**Q2**	**Q3**	**Q4**	
**Obesity**					
Intake (mg/d)	234.71 ± 126.58	348.41 ± 127.89	479.57 ± 134.04	747.18 ± 181.76	
Case/*N*	88/1,385	97/1,385	75/1,385	77/1,385	
Model 1^a^	Reference	1.06 (0.80–1.42)	0.77 (0.57–1.06)	0.80 (0.59–1.08)	0.050
Model 2^b^	Reference	0.93 (0.70–1.25)	0.66 (0.48–0.90)	0.63 (0.47–0.87)	0.001
Model 3^c^	Reference	0.93 (0.70–1.25)	0.65 (0.47–0.89)	0.63 (0.46–0.85)	0.001
**Hypertension**					
Intake (mg/d)	230.67 ± 125.54	243.58 ± 130.47	473.72 ± 132.59	740.52 ± 180.90	
Case/*N*	306/1,334	302/1,332	302/1,334	318/1,333	
Model 1	Reference	0.99 (0.84–1.16)	0.89 (0.76–1.04)	0.95 (0.81–1.11)	0.352
Model 2	Reference	0.93 (0.78–1.10)	0.80 (0.66–0.92)	0.84 (0.71–0.99)	0.024
Model 3	Reference	0.92 (0.78–1.09)	0.77 (0.65–0.92)	0.82 (0.69–0.97)	0.012
**Type 2 diabetes**					
Intake (mg/d)	213.90 ± 72.55	367.48 ± 34.49	494.17 ± 39.52	726.18 ± 132.12	
Case/*N*	117/1,554	119/1,554	132/1,554	133/1,554	
Model 1	Reference	1.01 (0.78–1.30)	1.04 (0.81–1.34)	1.04 (0.81–1.34)	0.700
Model 2	Reference	0.97 (0.74–1.28)	1.03 (0.79–1.34)	0.93 (0.71–1.21)	0.609
Model 3	Reference	0.96 (0.72–1.26)	1.02 (0.78–1.33)	0.95 (0.73–1.24)	0.759
**Cardiovascular disease**					
Intake (mg/d)	233.57 ± 126.05	343.01 ± 129.64	478.79 ± 134.70	746.91 ± 183.90	
Case/N	39/1,562	42/1,561	35/1,562	34/1,561	
Model 1	Reference	1.05 (0.68–1.62)	0.80 (0.50–1.26)	0.77 (0.49–1.23)	0.166
Model 2	Reference	0.95 (0.60–1.50)	0.70 (0.43–1.13)	0.68 (0.42–1.09)	0.065
Model 3	Reference	0.87 (0.55–1.39)	0.66 (0.40–1.06)	0.67 (0.41–1.09)	0.072

a *Model 1 adjusted with age and gender*.

b *Model 2 adjusted with age, gender, BMI, alcohol consumption rate, smoking rate, physical activity, and education*.

c *Model 3 adjusted with age, gender, BMI, alcohol consumption rate, smoking rate, physical activity, education, dietary energy intake, and AHEI. BMI, body mass index; AHEI, alternative healthy eating index*.

### Association Between Dietary Sources of Calcium With Obesity and Hypertension

**Figure 2:**
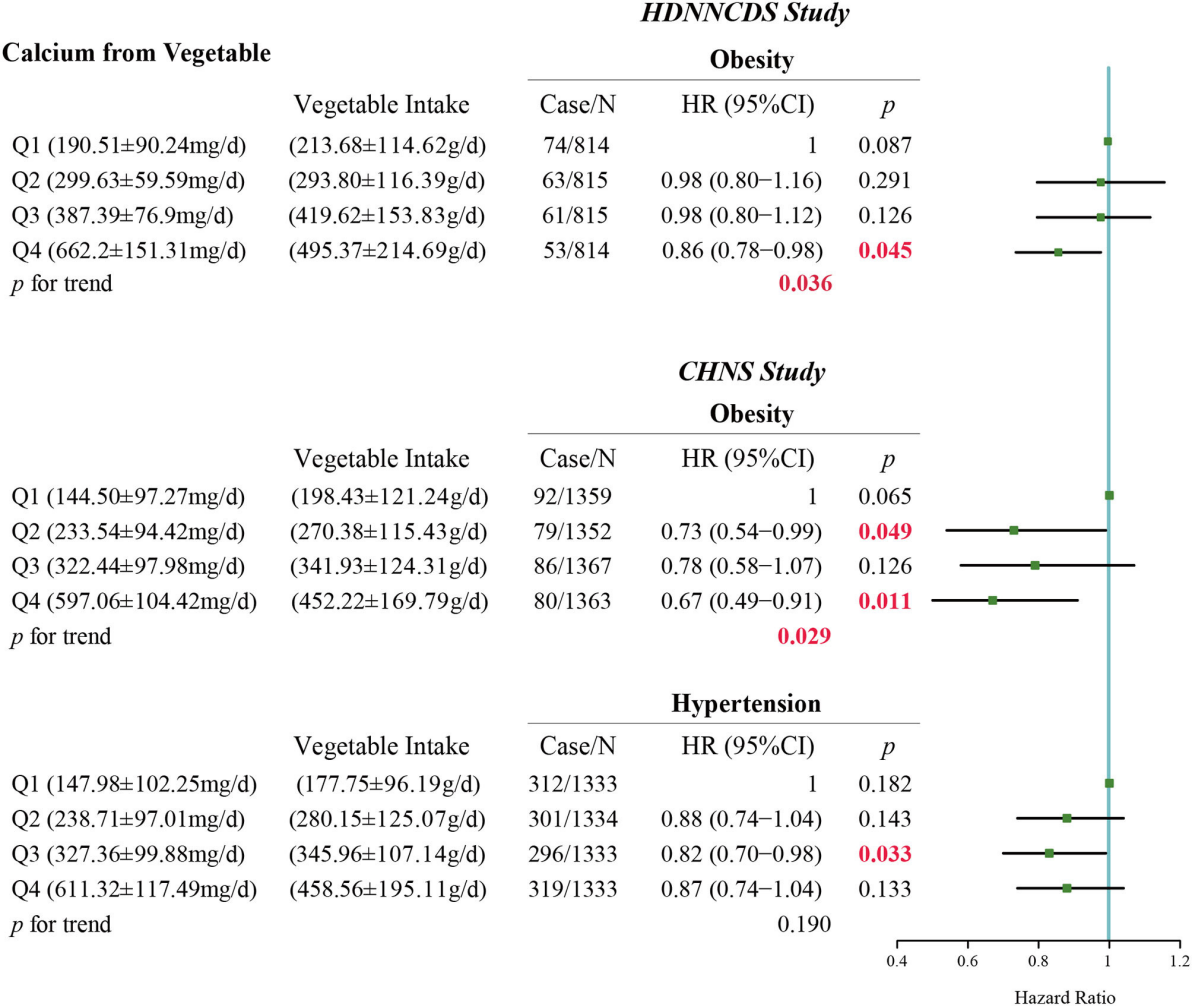
Association between calcium intake from vegetable with the risks of obesity and hypertension in HDNNCDS study and CHNS study. Models adjusted with age, gender, body mass index, alcohol consumption rate, smoking rate, physical activity, education, dietary energy intake, and alternative healthy eating index.

According to the dietary surveys, the main food sources of dietary calcium were grain, beans, vegetables, fruit, meat, dairy, eggs, and seafood in HDNNCDS and CHNS. After adjusting potential confounders and related dietary factors, in HDNNCDS, we found that dietary calcium from the vegetable source was negatively associated with the risk of obesity (*p* for trend = 0.036) (Figure 2). In the multivariate-adjusted model, the hazard risk of the highest quartile of calcium intake from vegetables was 0.86 (95% CI: 0.78–0.98, *p* = 0.045). Likewise, in CHNS, dietary calcium from the vegetable source was associated with a depressed incidence of obesity (*p* for trend = 0.029, HR of highest quartile: 0.67, 95% CI: 0.49–0.91, *p* = 0.011). For hypertension in CHNS, we did not find a significant linear trend between calcium from the vegetable source and the incidence of hypertension (*p* for trend = 0.190). However, the hazard ratio of hypertension in the third quartile of dietary calcium from vegetables relative to that in the lowest quartile was 0.82 (95% CI: 0.70–0.98, *p* = 0.033). After further adjusting the vegetable intake in the model, the relationship between dietary calcium intake and obesity and hypertension was not significant (*p* for trend = 0.084 and 0.444 for obesity in HDNNCDS and CHNS, respectively; *p* for trend = 0.221 for hypertension in CHNS) (Table 5). Otherwise, Table S1 shows the differences in incidences of obesity and hypertension between people who consumed and did not consume fruit or dairy in CHNS. We found that people who consumed fruit had a significantly lower incidence of hypertension than people who did not consume fruit (20.0% vs. 23.5%, *p* = 0.008). In addition, we did not find significant associations between other food sources of dietary calcium with the incidences of obesity and hypertension in HDNNCDS and CHNS (Supplementary Figures S5, S6).

**Table 5:** Hazard ratios (HRs) and 95% CIs of obesity and hypertension according to energy-adjusted dietary calcium intake in HDNNCDS and CHNS.

**Variables**	**Energy-adjusted dietary calcium intake**				***p_trend_***
	**Q1**	**Q2**	**Q3**	**Q4**	
**HDNNCDS**					
**Obesity**					
Case/*N*	176/1,650	112/1,654	126/1,652	92/1,650	
Multivariate-adjusted^a^	Reference	0.65 (0.46–0.93)	0.76 (0.54–1.07)	0.58 (0.40–0.83)	0.015
Added adjusted for vegetable intake	Reference	0.79 (0.52–1.18)	0.73 (0.48–1.10)	0.67 (0.42–1.08)	0.084
**CHNS**					
**Obesity**					
Case/*N*	88/1,385	97/1,385	75/1,385	77/1,385	
Multivariate-adjusted	Reference	0.93 (0.70–1.25)	0.65 (0.47–0.89)	0.63 (0.46–0.85)	0.001
Added adjusted for vegetable intake	Reference	1.04 (0.77–1.40)	0.80 (0.58–1.11)	0.92 (0.64–1.32)	0.444
**Hypertension**					
Case/*N*	306/1,334	302/1,332	302/1,334	318/1,333	
Multivariate-adjusted	Reference	0.92 (0.78–1.09)	0.77 (0.65–0.92)	0.82 (0.69–0.97)	0.012
Added adjusted for vegetable intake	Reference	1.01 (0.85–1.20)	0.93 (0.77–1.11)	1.15 (0.94–1.40)	0.221

a *Multivariate-model adjusted with age, gender, BMI, alcohol consumption rate, smoking rate, physical activity, education, dietary energy intake and AHEI. BMI, body mass index; AHEI, alternative healthy eating index*.

## Discussion

In HDNNCDS and CHNS, we found dietary calcium was inversely associated with the incidence of obesity; nevertheless, there was no correlation between dietary calcium intake and bone density, and also the incidence of T2DM and CVD. Meanwhile, the relationship between dietary calcium intake and the risk of hypertension in the two cohorts was inconsistent.

Dietary calcium intakes were 484.32 ± 198.61 and 451.35 ± 203.56 (mean ± SD) mg/d in HDNNCDS and CHNS, respectively. According to the survey, in 2012, the average daily calcium intake of Chinese residents was 366.1, 412.4 mg in urban areas, and 321.4 mg in rural areas (21). These were similar to the mean dietary calcium in the two cohort surveys in our study, both well below the recommended calcium intake of 800 mg/d, while it must be noted that Chinese dietary reference intakes for calcium were largely based on calcium balance data and clinical trials derived from the developed country (22). Measures of calcium balance have limitations and their precision is difficult to ascertain (23). Ideally, each nation should establish its own RDA of calcium based on the ethnic make-up of its population (24). Due to different dietary cultures, genetics, lifestyles, and food production histories, environments, despite the adjustment of sizes, the recommended calcium intake in western countries may not be entirely applicable to Chinese (25, 26). Meanwhile, a meta-analysis for Chinese adults of calcium balance studies found across all the Chinese balance studies, after adjustment for calcium intake, region, gender, diet, adaptation duration, and balance period, calcium intake equaled calcium output at intakes of 400–500 mg/d (9). It was suggested that the current calcium recommended intake based on western countries may not be suitable for the Chinese population.

In HDNNCDS, after adjusting the covariates, the relationship between dietary calcium intake and BMD was not statistically significant. Our finding appears to be consistent with the results from studies in premenopausal women showing that daily dietary calcium intake did not correlate with BMD (27). Moreover, a number of the high-quality meta-analyses that found calcium supplementation alone or with vitamin D were not associated with reduced fracture incidence among community-dwelling adults (28, 29). One possible explanation for this study is that calcium intake has already met the bone requirements in the lowest quantile group, so increasing calcium intake cannot continue to increase bone density.

Apart from that, we used the same statistical model to study the relationship between dietary calcium and disease in two large prospective cohorts of the same race. The results indicated that the association between dietary calcium intake and the risk of obesity was significant in both HDNNCDS and CHNS. To enhance the credibility of the results, in our study, stratified analyses were performed based on age (Supplementary Figures S2, S3) and serum 25OHD_3_ levels (Figure S4). Interestingly, we found that this kind of protective relationship exists only in middle-aged people and is independent of serum vitamin D levels. In a cohort study of middle-aged women, the decrease in annual average weight gain was related to the increase in calcium intake (30). Epidemiologic studies based on the National Health and Nutrition Examination Study III (NHANES III) also have found that there was a reduction in risk for obesity with each increasing quartile of calcium intake (31).

However, it is worth noting that Chinese people live on plant-based diets poor in calcium quantity. If residents rely on the traditional Chinese diet, it is hard to reach 800 mg/d calcium intake, unless calcium supplements are used. Although milk and dairy products are the main sources of calcium (32), the Chinese intake of calcium from eggs, fish, shrimp, and milk is <5%, whereas vegetables, cereals, and legumes are the main sources of dietary calcium (28, 33). However, more than 80% of dietary calcium in westerners was derived from dairy and meat products (34). To explore the relationship between major food sources of dietary calcium and chronic diseases, we further analyzed the association of calcium intake in vegetables, fruit, dairy, and other foods with the incidences of obesity and hypertension. We found that only calcium consumption in vegetables was consistent with total calcium intake, that the higher the calcium intake in vegetables, the lower the risk of obesity and hypertension. Simultaneously, as calcium consumption in vegetable increases, the intake of calcium and vegetables increased together, which possibly contributes to a protective effect on the risk of obesity and hypertension. Therefore, in other to exclude the influence of vegetable intake on the relationship between calcium intake and obesity and hypertension, we further adjusted the vegetable intake in the model. We found that the relationship between dietary calcium intake and obesity and hypertension was strongly attenuated, after further adjustment for the intake of vegetables. Besides, we divided the participants into two groups according to the energy-adjusted vegetable consumption level. We separately analyzed the relationship between dietary calcium and obesity at different levels in the two cohorts (Figure S7). We found that dietary calcium was still negatively correlated with obesity risk at higher vegetable consumption levels, but there was no statistically significant relationship between dietary calcium and obesity when vegetable consumption was lower. In any case, such results indicated although the association between dietary calcium intake and obesity and hypertension has been found, it is probably due to the beneficial effects of vegetables on body weight and blood pressure (35–37). Besides, due to the difference in the research methods of the dietary surveys in the two cohort studies, FFQ in HDNNCDS and 3-day 24-h retrospective in CHNS respectively, which led to the intake of dairy and fruits as the main dietary sources of calcium in CHNS, may be underestimated. Since fewer people consumed fruits and dairy products, we compared the incidence of obesity or hypertension in people who consumed fruit and dairy with those who did not (Table S1). Even though we found a significantly different incidence of hypertension between the people who consumed fruit and those who did not in CHNS, owing to the number of people who consume fruit is too small, accounting for only 14% of the total number of people, the statistically significant difference between the incidence rates has no actual clinical significance.

To the best of our knowledge, this study is the first attempt to comprehensively explore the relationship between different calcium intake and health in Chinese residents in both a community prospective cohort and a national prospective cohort. We used the same statistical model to study the relationship between dietary calcium and disease in two large prospective cohorts of the same race and reached similar conclusions, which increased the credibility of our findings. In addition, this study also adopted two dietary survey methods to assess dietary calcium intake, with one being relatively accurate (24 h recorded), and another reflecting long-term dietary habits (FFQ), and found no significant correlation between dietary calcium and the risk of chronic diseases. Besides, we measured serum 25OHD_3_ levels based on UPLC, enhancing our ability to entirely evaluate the associations among calcium, vitamin D, and chronic diseases. Nevertheless, there are also some limitations to our study. In the CHNS, CVD is diagnosed by self-reported information of the history of stroke and/or myocardial infarction, and there is no detection of related metabolic indicators. Although this is one of the limitations of the CHNS survey, this standard has also been used in previous articles about CVD research. Additionally, although we have adjusted for many potential confounders including demographic, lifestyle, and diet, our results were also limited by the possibility of unaccounted for confounders.

## Conclusions

In conclusion, this study explored the relationship between calcium intake and the health of Chinese residents from different angles. Although according to our results, the current calcium intake of Chinese residents is negatively correlated with obesity and high blood pressure, the relationship between them is unstable and may be related to the intake of vegetables. Furthermore, we demonstrated that an additional increase in dietary calcium intake may not have significant benefits for bone health, type 2 diabetes, and CVD, on the premise that the calcium intake of Chinese people is generally lower than the recommended intake. Based on our findings to date, although the current calcium intake of Chinese residents is lower than the recommended intake, the calcium concentration in the human body may have reached a balanced state and can meet the needs of the body. It is, thus, not reasonable to emphasize additional calcium supplementation.

## Data Availability Statement

For HDNNCDS, the anonymized raw data supporting the conclusions of this article can be obtained upon reasonable request to the corresponding author. For CHNS, the dataset presented in this study can be found in online repositories. The name of the repository/repositories and accession number(s) can be found here: https://www.cpc.unc.edu/projects/china.

## Ethics Statement

Written informed consent was obtained from the individual(s) for the publication of any potentially identifiable images or data included in this article.

## Author Contributions

YL, CS, and XM contributed to the design and conduct of the research. XG and JG carried out data analysis and the initial draft of the paper. JW, ZZ, QS, and KH conducted the data collection and advised on statistical analysis. All authors reviewed and edited the draft, and approved the final version of the manuscript.

## Funding

This work was supported by the ZhenDong Nutrition Research Fund of Chinese Nutrition Society (CNS-ZD2020-71).

## Conflict of Interest

The authors declare that the research was conducted in the absence of any commercial or financial relationships that could be construed as a potential conflict of interest.

## Publisher's Note

All claims expressed in this article are solely those of the authors and do not necessarily represent those of their affiliated organizations, or those of the publisher, the editors and the reviewers. Any product that may be evaluated in this article, or claim that may be made by its manufacturer, is not guaranteed or endorsed by the publisher.
