# Supplementary material for: Association of Dietary Calcium Intake With Bone Health and Chronic Diseases: Two Prospective Cohort Studies in China
Source: Front Nutr. 2021 Dec 24;8:683918. doi: 10.3389/fnut.2021.683918 (PMC8740131; doi:10.3389/fnut.2021.683918)
Supplement: Supplementary file 1 [file Data_Sheet_1.docx]

Supplementary Material


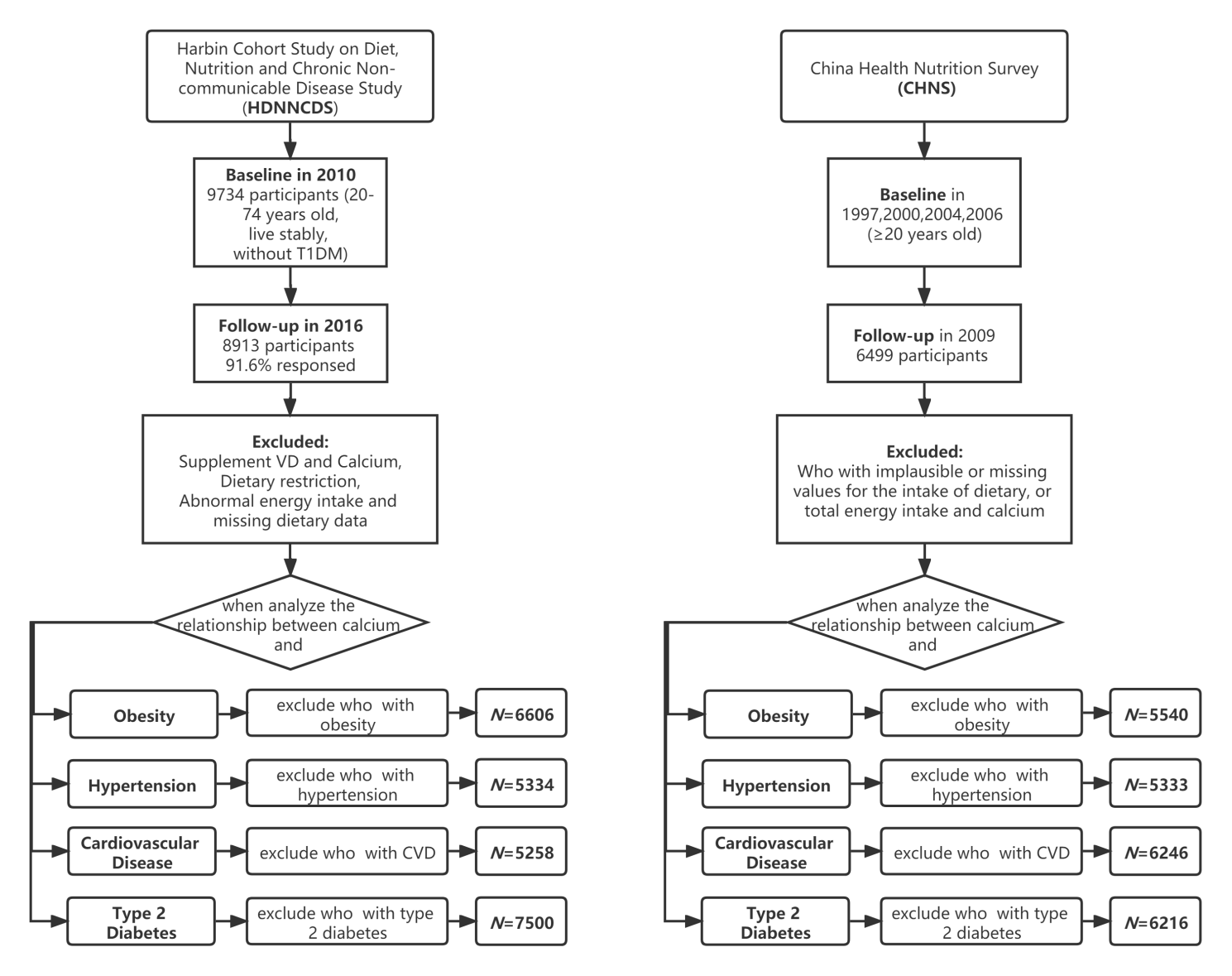


**Supplementary Figure 1.** Study flowchart on selection of the participant.


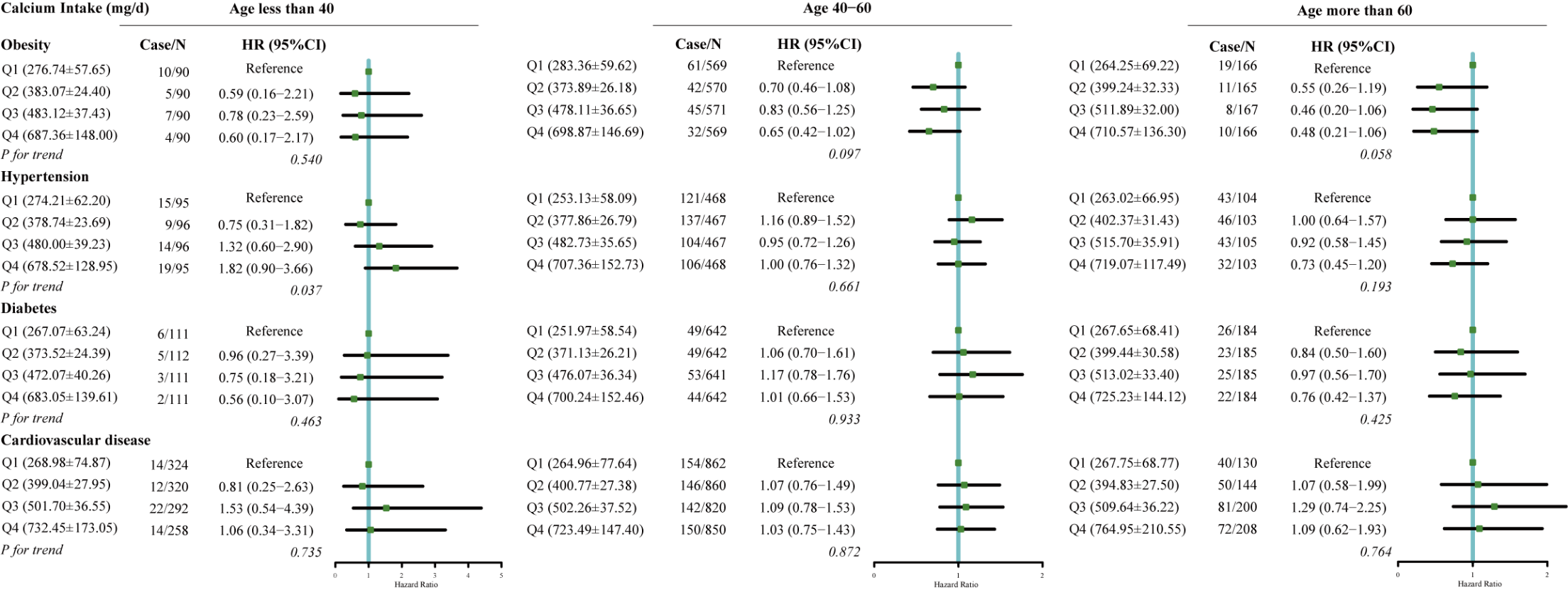


**Supplementary Figure 2.** Association between dietary calcium intake and the risks of chronic diseases according to age in HDNNCS study. Models adjusted with age, gender, body mass index, alcohol consumption rate, smoking rate, physical activity, education, dietary energy intake and Alternative healthy eating index.


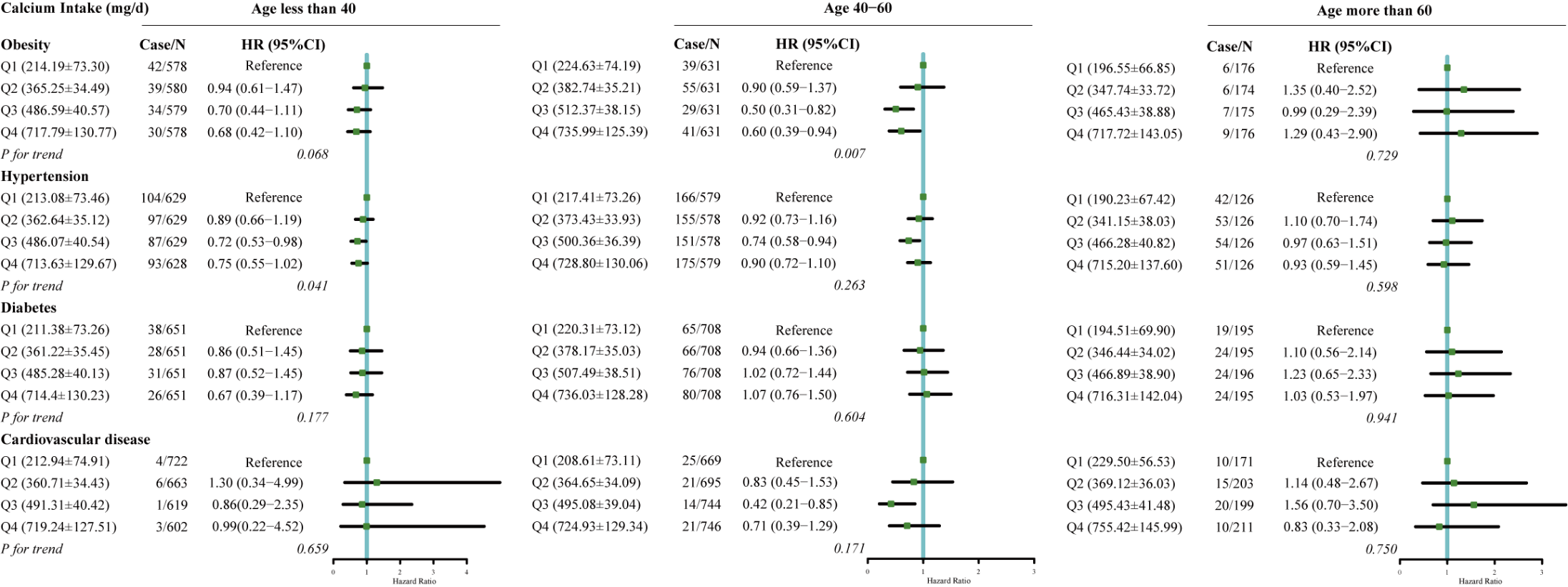


**Supplementary Figure 3.** Association between dietary calcium intake and the risk of chronic diseases according to age in CHNS study. Models adjusted with age, gender, body mass index, alcohol consumption rate, smoking rate, physical activity, education, dietary energy intake and Alternative healthy eating index.


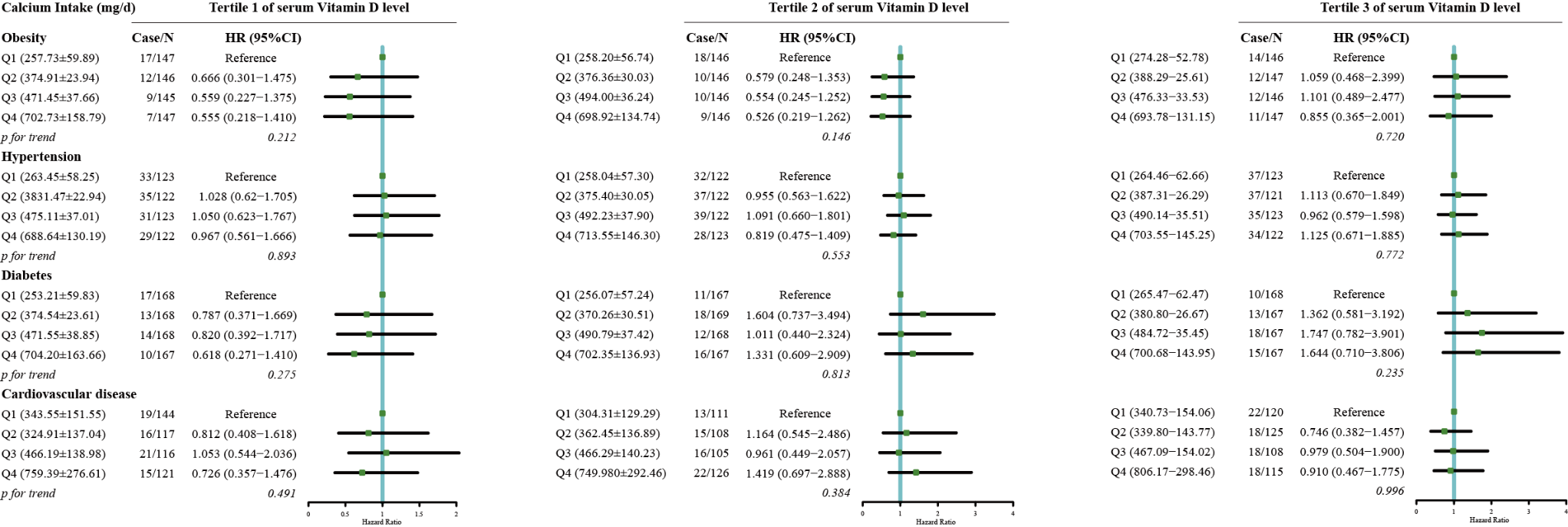


**Supplementary Figure 4.** Association between dietary calcium intake and the risks of chronic diseases according to serum Vitamin D level in HDNNCS study. Models adjusted with age, gender, body mass index, alcohol consumption rate, smoking rate, physical activity, education, dietary energy intake and Alternative healthy eating index.


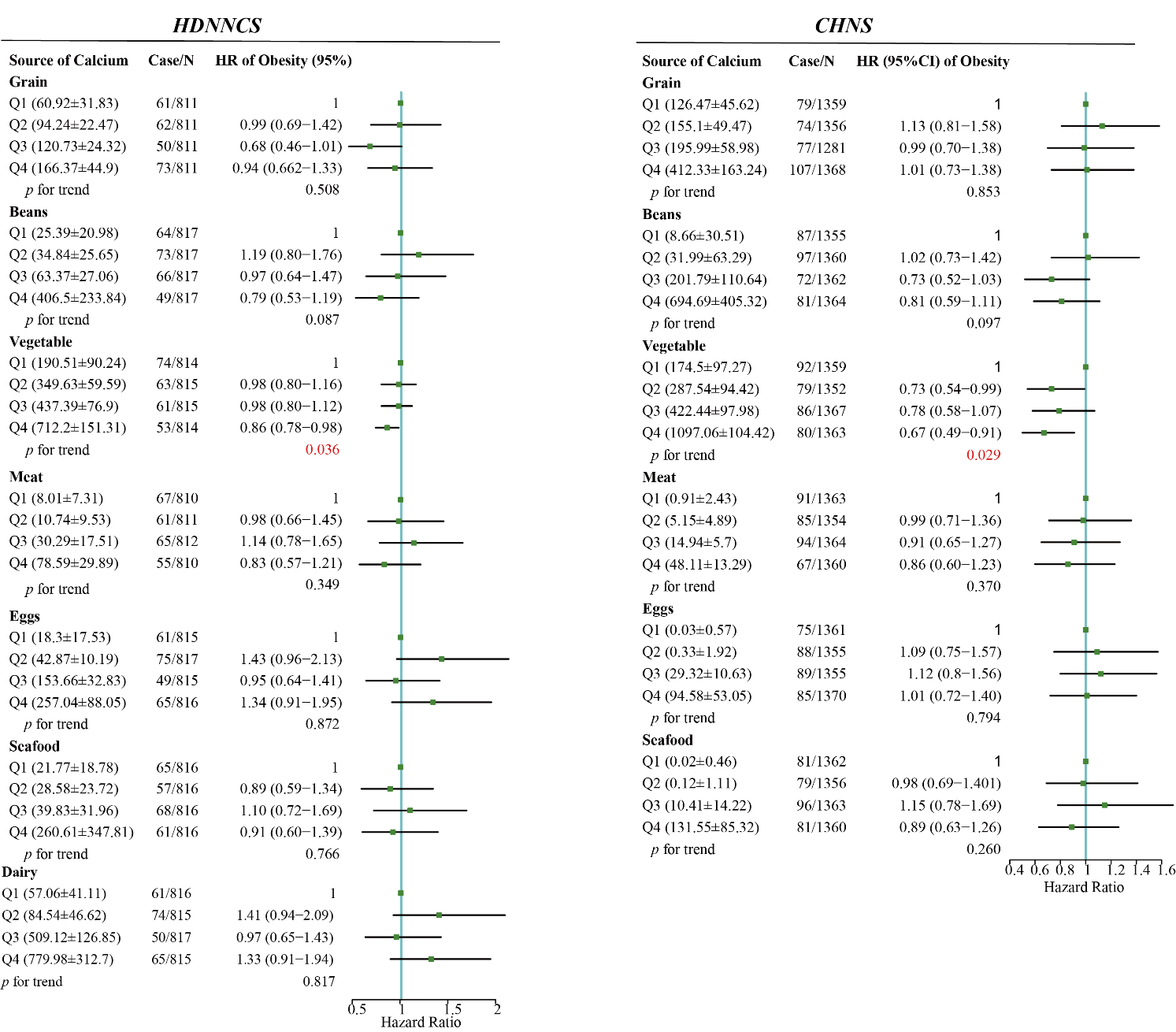


**Supplementary Figure 5**. Association between food sources of dietary calcium and the risks of obesity in HDNNCS and CHNS study. Models adjusted with age, gender, body mass index, alcohol consumption rate, smoking rate, physical activity, education, dietary energy intake and Alternative healthy eating index.


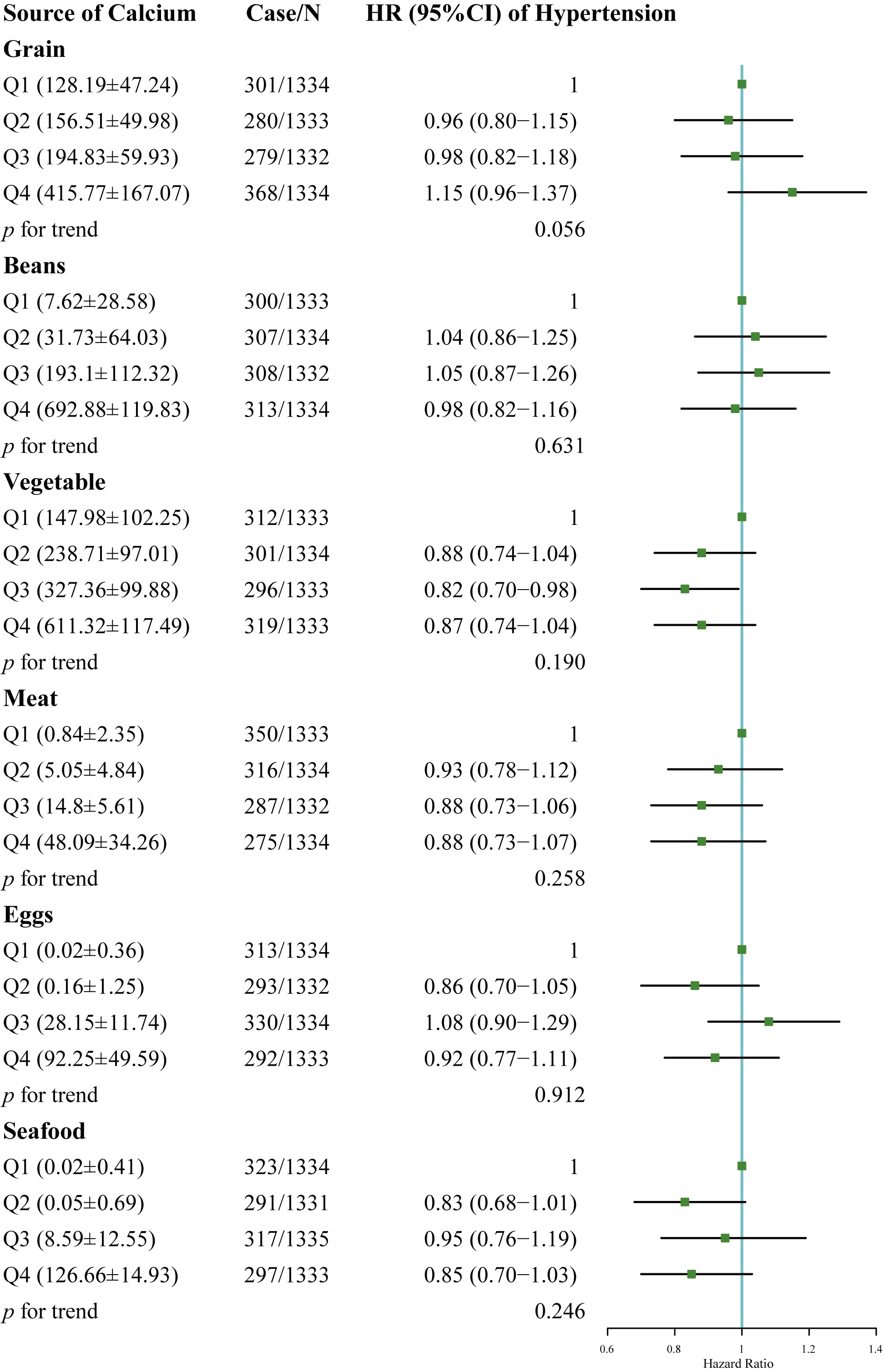


**Supplementary Figure 6**. Association between food sources of dietary calcium and the risks of hypertension in CHNS study. Models adjusted with age, gender, body mass index, alcohol consumption rate, smoking rate, physical activity, education, dietary energy intake and Alternative healthy eating index.


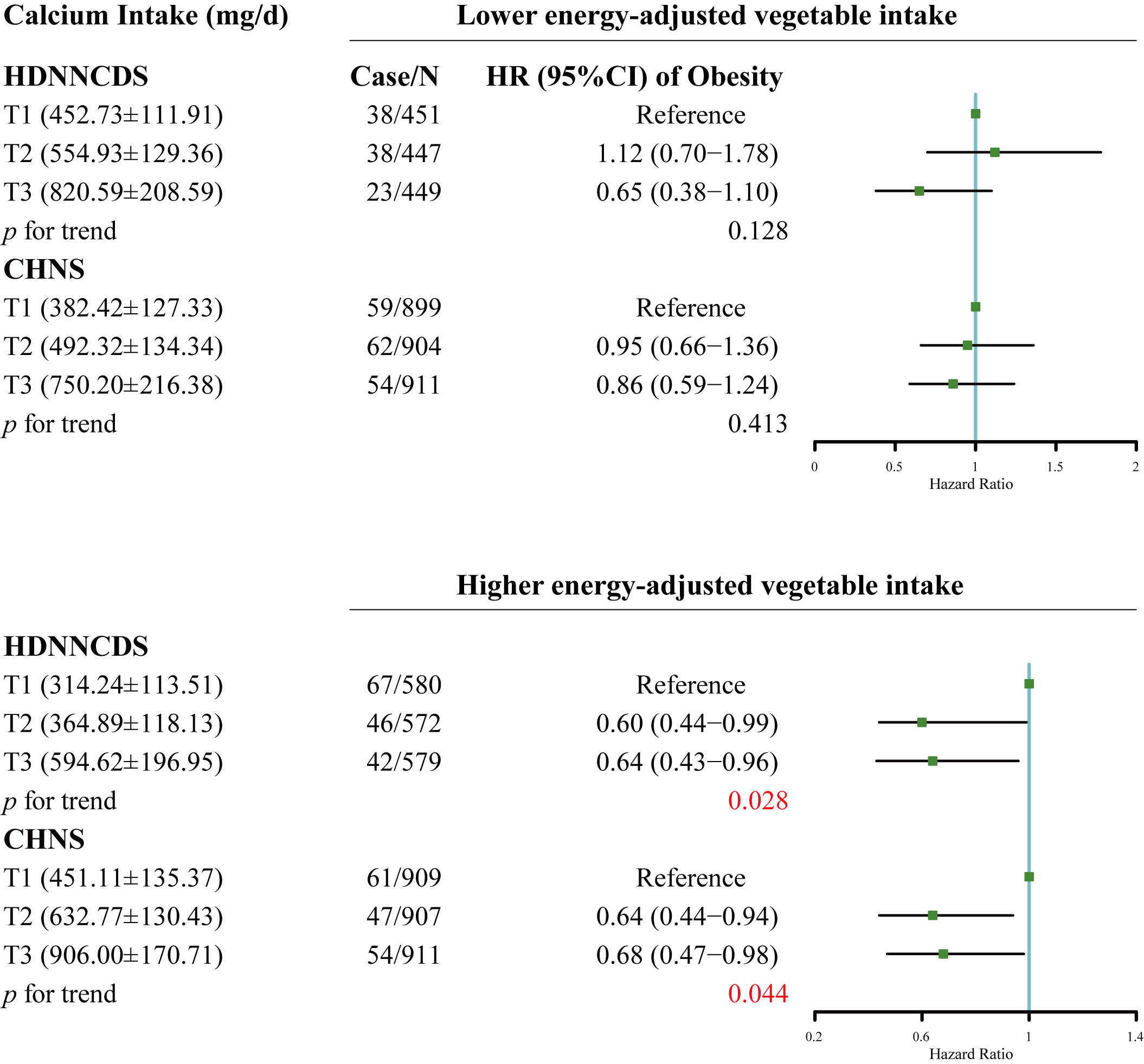


**Supplementary Figure 7**. Association between dietary calcium and the risk of obesity in different levels of energy-adjusted vegetable consumption. Models adjusted with age, gender, body mass index, alcohol consumption rate, smoking rate, physical activity, education, dietary energy intake and Alternative healthy eating index.

**Supplementary Table 1** Differences in incidences of obesity and hypertension between people who consumed and did not consume fruit or dairy in CHNS.^a^

| **Dietary calcium consumption** | | **Obesity (n=5441)** | | | **Hypertension (n=5333)** | | |
| --- | --- | --- | --- | --- | --- | --- | --- |
|  |  | N | Incidence | *P^b^* | N | Incidence | *p* |
| **From fruit** | Consumed | 786 | 6.4% | 0.932 | 746 | 20.0% | **0.008** |
|  | Non-consumed | 4655 | 6.0% |  | 4587 | 23.5% |  |
| **From dairy** | Consumed | 210 | 6.1% | 0.341 | 185 | 19.5% | 0.257 |
|  | Non-consumed | 5231 | 6.5% |  | 5148 | 23.2% |  |

*^a^*Incidences were compared between subjects who consumed fruit or dairy and those who did not consume by using univariate logistic regression models.

*^b^*Models adjusted with age, gender, body mass index, alcohol consumption rate, smoking rate, physical activity, education, dietary energy intake and Alternative healthy eating index.
